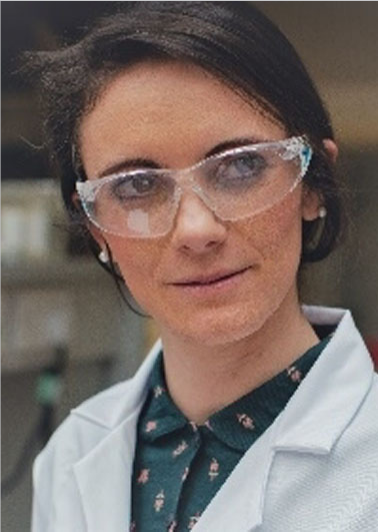# Frustrated Radical Pairs: Insights from EPR Spectroscopy

**DOI:** 10.1002/anie.202010633

**Published:** 2020-11-17

**Authors:** Ayan Dasgupta, Emma Richards, Rebecca L. Melen

**Affiliations:** ^1^ School of Chemistry Cardiff Catalysis Institute Cardiff University Main Building, Park Place Cardiff CF10 3AT UK

**Keywords:** electron paramagnetic resonance, frustrated Lewis pair, main group chemistry, radicals, single-electron transfer

## Abstract

Progress in frustrated Lewis pair (FLP) chemistry has revealed the importance of the main group elements in catalysis, opening new avenues in synthetic chemistry. Recently, new reactivities of frustrated Lewis pairs have been uncovered that disclose that certain combinations of Lewis acids and bases undergo single‐electron transfer (SET) processes. Here an electron can be transferred from the Lewis basic donor to a Lewis acidic acceptor to generate a reactive frustrated radical pair (FRP). This minireview aims to showcase the recent advancements in this emerging field covering the synthesis and reactivities of frustrated radical pairs, with extensive highlights of the results from Electron Paramagnetic Resonance (EPR) spectroscopy to explain the nature and stability of the different radical species observed.

## Graphical abstract

**Single or Double?** This Minireview highlights the recent new reactivities of frustrated Lewis pairs that disclose that certain combinations of Lewis acids and bases undergo single‐electron transfer processes. In these studies, Electron Paramagnetic Resonance spectroscopy has been instrumental in the elucidation of reaction pathways.

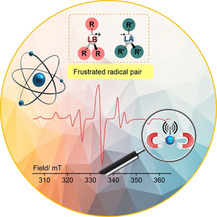

## 1 Introduction

**Scheme 1:**
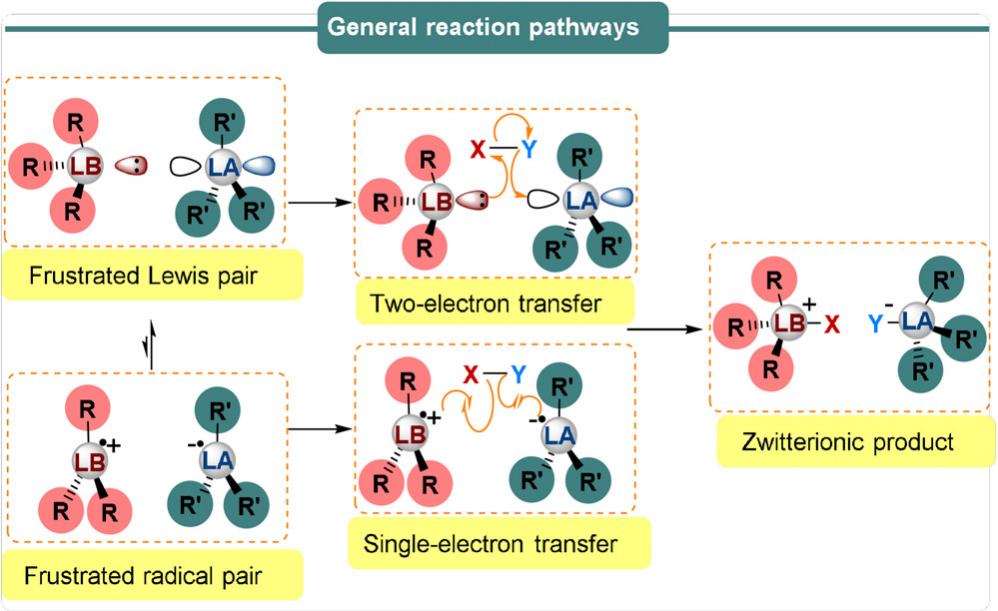
Generic representation of the reactivity of FLPs and FRPs in the activation of small molecules.

The combination of a Lewis acid (LA) and a Lewis base (LB) bearing sterically encumbered groups leads to the formation of frustrated Lewis adducts in which the unquenched reactivities of Lewis acidic and basic sites are capable of reversibly activating H_2_.^[1]^ The seminal work from Stephan and co‐workers^[2]^ in 2006 demonstrated that a metal‐free compound Mes_2_P(C_6_F_4_)B(C_6_F_5_)_2_ was active in reversible H_2_ binding. The donor–acceptor ability of archetypal frustrated Lewis pairs (FLPs) makes them competitive with transition metal catalysts towards H_2_ heterolysis. Since its inception in 2006, a range of FLPs are now known in the literature,^[1]^ comprising a variety of different Lewis acidic and basic components, including transition metal^[3]^ or chiral components,^[4]^ that may be both intramolecular or intermolecular. Vast efforts have already been made to demonstrate the further applications of FLPs towards the activation of other small molecules including olefins, alkynes, CO_2_, SO_2_, N_2_O, and NO.^[5]^ Extensive studies later disclosed that FLPs can be used as alternatives to transition metal systems and have successfully been employed for a plethora of organic transformations.^[6]^ Until recently, it was believed that the mode of small molecule activation was a heterolytic process.^[7]^ However, more recently it has been observed that certain combinations of Lewis acid and Lewis base enable competitive donation of a *single* electron from the donor Lewis base to the empty p‐orbital of the acceptor Lewis acid to afford a frustrated radical pair (FRP).[^5a^, ^8^] This phenomenon was in sharp contrast with the conventional FLP mechanism, wherein the donor Lewis base donates *two* electrons to the H–X σ*‐orbital, followed by heterolytic cleavage of the H–X σ‐bond and subsequent donation of *two* electrons to the empty p‐orbital of the acceptor Lewis acid (Scheme 1). Conversely, FRPs are proposed to cleave the H–X bond in a homolytic fashion. This minireview aims to highlight this new direction of single‐electron reactivity in FLP chemistry with recent examples of different LB/LA combinations including phosphorus–alane (P/Al), phosphorus–borane (P/B), nitrogen–borane (N/B) and carbon–borane (C/B) FRPs. In particular, we will focus on results from Electron Paramagnetic Resonance (EPR) spectroscopy, which has been instrumental in the elucidation of alternative single‐electron transfer (SET) reaction pathways to highlight the unusual reactivities and stabilities of frustrated radical pairs.

## 2 Group 13/15 Frustrated Lewis Pairs

**Figure 1:**
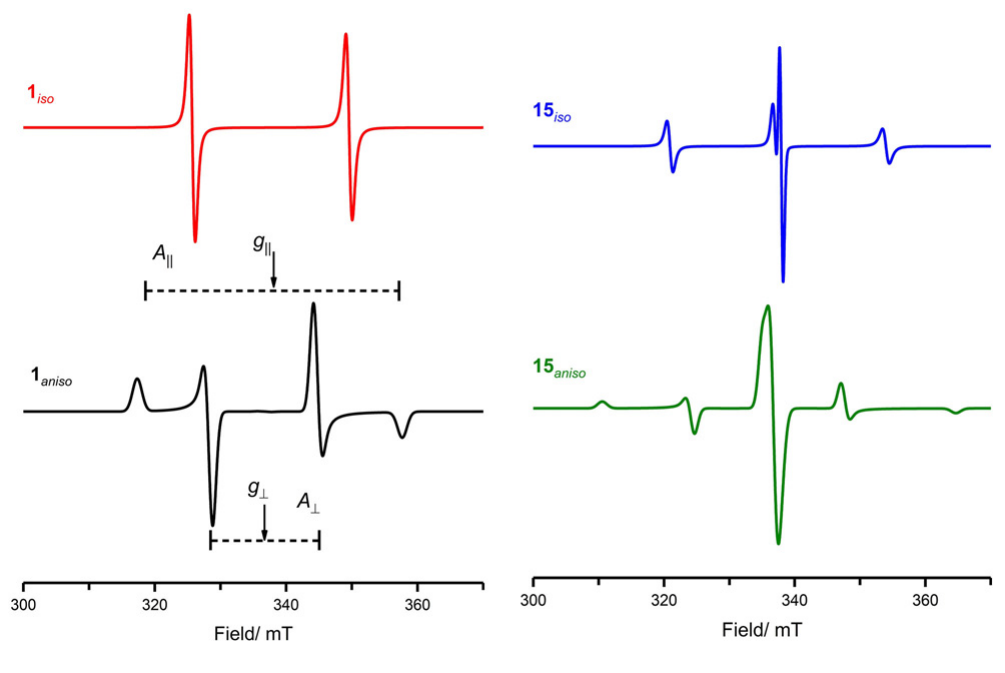
Isotropic and anisotropic EPR spectra of Mes_3_P^.+^ (**1** left), and (Mes_2_P)_2_^.+^ (**15** right), simulated using data reported in Table 1.

**Scheme 2:**
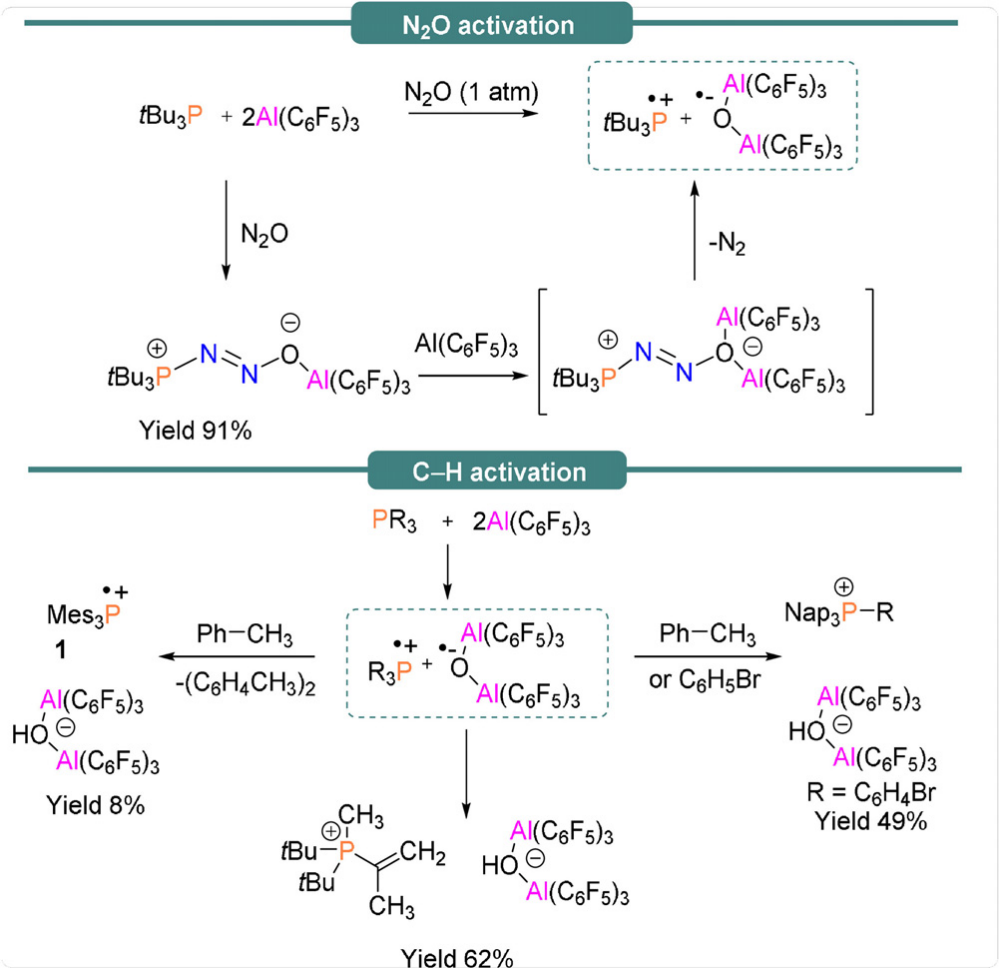
P/Al FLP‐mediated N_2_O and C–H activation of toluene and bromobenzene.

At the heart of radical formation within FLPs is the backbone of the participating Lewis acid and Lewis base. In this section, we will discuss FLPs that comprise a Group 15 (phosphorus or nitrogen) Lewis base and a Group 13 (boron or aluminium) Lewis acid which have been found to undergo single‐electron transfer reactions. The first example of a proposed SET mechanism using phosphorus/boron‐based FLPs was suggested by Piers and co‐workers in 2011.^[9]^ The activation of dihydrogen via four plausible mechanisms (including homolytic and heterolytic H−H bond cleavage) using a *t*Bu_3_P/B(C_6_F_5_)_3_ FLP system was proposed. Single‐electron oxidation of a Lewis base by a borane component is notionally feasible and would lead to the formation of a radical ion pair (Table 1); however, based on the reduction potential of B(C_6_F_5_)_3_ (−1.17 V vs. Cp_2_Fe^0/+^ in THF),^[10]^ and the oxidation potential of *t*Bu_3_P (0.90 V vs. Cp_2_Fe^0/+^ in MeCN)^[11]^ (Table 2), the concentration of the radical ion Lewis pair is expected to be insignificant in comparison to the Lewis pair responsible for heterolytic dihydrogen cleavage. Shortly after this report, Stephan and co‐workers^[12]^ noticed that FLPs bearing the more Lewis acidic alane [Al(C_6_F_5_)_3_] and *t*Bu_3_P react readily with N_2_O (1 atm) to afford *t*Bu_3_P(N_2_O)Al(C_6_F_5_)_3_ (yield 91 %). This complex further reacts with an additional equivalent of Al(C_6_F_5_)_3_ and releases N_2_ to generate a proposed transient FRP [*t*Bu_3_P]^.+^[(μ‐O)(Al(C_6_F_5_)_3_)_2_]^.−^ (Scheme 2, top). C−H bond activation of one of the *tert‐*butyl groups affords the salt [*t*Bu_2_PMe(C(CH_2_)Me)][(μ‐OH)(Al(C_6_F_5_)_3_)_2_] (yield 62 %) as the product (Scheme 2, bottom). Alternatively, if Mes_3_P is allowed to react with [Al(C_6_F_5_)_3_]⋅tol in toluene, C−H bond activation of the solvent is observed affording [Mes_3_P]^.+^[(μ‐OH)(Al(C_6_F_5_)_3_)_2_]^−^. While EPR studies on the reactive intermediate [R_3_P]^.+^[(μ‐O)(Al(C_6_F_5_)_3_)_2_]^.−^ were not undertaken, the solution‐phase X‐band EPR spectrum of the dissolved crystals of the product [Mes_3_P]^.+^[(μ‐OH)(Al(C_6_F_5_)_3_)_2_]^−^ in bromobenzene revealed a doublet (*g*_iso_=2.0056, *a*_iso_(^31^P)=239 G (670 MHz)) for the suggested generation of the phosphonium radical cation, Mes_3_P^.+^ (**1**) (Figure 1 and Table 1). Alternatively when Nap_3_P (Nap=naphthyl) was employed in the reaction, C–H activation of the solvent (toluene or bromobenzene) led to [(Nap)_3_PR][(μ‐OH)(Al(C_6_F_5_)_3_)_2_] (R=CH_2_Ph, C_6_H_4_Br) (Scheme 2, bottom). A few years later, Stephan et al.^[13]^ reported distinctly different reaction pathways when FLPs, *t*Bu_3_P/E(C_6_F_5_)_3_ and Mes_3_P/E(C_6_F_5_)_3_ (E=B, Al), were employed in the reaction with *p*O_2_C_6_Cl_4_ and Ph_3_SnH.

**Table 1:** Spin Hamiltonian parameters for radical species generated during FLP reactions.^[a]^

Radical	*g*_iso_	*a*_iso_ [MHz]^[a,b]^	Reference
Phosphorus			
Mes_3_P^.+^ (**1**)	2.012; *g*_∥_=2.010; *g*_⊥_=2.013	678; *A*_∥_=1135; *A*_⊥_=450	[18, 37]
*t*Bu_3_P^.+^ (**6**)	2.0047; *g*_∥_=2.0012; *g*_⊥_=2.0065	842; *A*_∥_(^31^P)=1365; *A*_⊥_(^31^P)=580	[14]
(Mes_2_P)_2_^.+^ (**15**)	2.014; *g*_∥_=2.009; *g*_⊥_=2.017	470; *A*_∥_=761; *A*_⊥_=325	[38, 39]
(Et_3_P)_2_^.+^	2.008; *g*_∥_=2.00; *g*_⊥_=2.012	1277; *A*_∥_=1511; *A*_⊥_=1160	[27]
(Bu_3_P)_2_^.+^	2.008; *g*_∥_=2.00; *g*_⊥_=2.012	1298; *A*_∥_=1540; *A*_⊥_=1177	[27]
Dipp_3_P^.+^	2.008	672	[37]
Tipp_3_P^.+^ (**14**)	*g*_∥_=2.002; *g*_⊥_=2.009	*A*_∥_=1168; *A*_⊥_=366	[37]
Xyl_3_P^.+^	2.0052	685	[37]
Nitrogen			
(*p*‐bromo‐N,N‐dimethylaniline)^.+^ (**8**)	2.0029	^14^N: 92.25; ^1^H_*methyl*_: 60.72; ^1^H_*o*_: 32.55; ^1^H_*m*_: 16.12	[27]
(4‐bromo‐*N*‐methyl‐*N*‐((trimethylsilyl) methyl)aniline)^.+^ (**8‐TMS**)	2.0033	^14^N: 23.1; ^1^H_*m*_: 3.73; ^1^H_*o*_: 9.68; ^1^H_*methylene*_: 27.8; ^1^H_*methyl*_: 20.7; ^29^Si: 8.78	[21]
(C(CH_3_)_2_C_6_H_3_)_3_N ^.+^ (**7**)	2.002	^14^N: 26.34; ^1^H_*3p*_: 8.52; ^1^H_*6m*_: 1.99	[21]
Boron			
B(C_6_F_5_)_3_^.−^ (**5**)	2.0114	B: 31; F_*6o*_: 12.94; F_*6m*_: 3.66; F_*3p*_: 14.9	[20]
K[Me_2_C(CONMes)_2_‐CC_6_F_4_‐BF(C_6_F_5_)_2_C^.^] (**9 a**)	2.022	B: 1.41; N_*2*_: 4.25; F_*2m*_: 5.66; F_*2o*_: 11.88	[22]
K[Me_2_C(CONMes)_2_‐CC_6_F_4_‐BH(C_6_F_5_)_2_C^.^] (**9 b**)	2.003	B: 0.82; N_*2*_: 4.53; F_*2m*_: 5.34; F_*2o*_: 11.02	[22]
K[Me_2_C(CONMes)_2_‐CC_6_F_4_‐B(OTf)(C_6_F_5_)_2_C^.^] (**9 c**)	2.003	B: 1.01; N_*2*_: 4.04; F_*2,1*_: 12.41. F_*2,2*_: 5.23; F_*4,3*_: 0.68; F_*2,4*_: 0.43	[22]
Me_2_C(CONMes)_2_‐CC_6_F_4_‐B(C_6_F_5_)_2_^.^ (**10**)	2.004	B: 1.46; N_*2*_: 2.61; F_*2,1*_: 14.16. F_*2,2*_: 2.94; F_*4,3*_: 4.48; F_*4,4*_: 0.09; F_*2,5*_: 1.98	[22]
Aluminium			
[(*t*Bu_2_MeSi)_3_Al]^.−^ (**2**)	2.005	Al: 173.99	[16]
Gallium			
[(*t*Bu_2_MeSi)_3_Ga]^.−^ (**3**)	2.015	^69^Ga: 346.89; ^71^Ga: 442.78	[16]
Germanium			
[BCHGe]^.+^ (**11**)	1.9881	^177, 179^Hf: 236.5	[23]
Carbon			
Ph_3_C^.^ (**12**)	1.999	H_*o*_: 7.29; H_*m*_: 3.08; H_*p*_: 7.83	[23]
[Me_2_C(CONMes)_2_‐CC_6_F_4_‐CPh_2_]^.+^ (**13**)	1.993	N_*2*_: 0.56; H_*2,1*_: 7.31; H_*2,2*_: 7.56; H_*4,3*_: 7.48; H_*4,4*_: 4.06; F_*2,5*_: 6.29	[22]
Di‐phenyl‐methylene	2.0030	H_*c*_: 23.43; H_*o,p*_: 8.55; H_*m*_: 3.42	[40, 41]
Fluorenyl (**4**)	2.002	H_*1*_: 38.96; H_*3*_: 11.15; H_*4*_: 2.55; H_*5*_: 10.54; H_*6*_: 1.79	[42]
Styryl	2.0023	H_*CH2*_: 116; H_*o,p*_: 17; 16.8	[43]
phenylacetylene	2.0021	H_*5*_: 7.71; H_*5*_: 2.38	[44]
Aminoxyl			
P/B‐FLP‐NO^.^ (**16**)	2.0089	^14^N: 18.5; ^31^P: 48.5; ^11^B: 9.1	[30]

[a] Subscript numbers are formatted as number of equivalent nuclei, followed by nucleus position, for example, F_*6o*_ reads 6 equivalent nuclei in the *ortho* position. [b] For conversion to field units, *a*/ mT=[10^9^ × (*h*/*g*μ_B_)] × *a*/ MHz, where *g*=*g*‐factor, *h*=Planck constant, μ_B_=Bohr magneton.

**Table 2:** Oxidation potentials, ionisation potentials and electron affinities for Lewis acids and Lewis bases

Substrates	Ionisation Potential [eV]	Conditions	Reference
Mes_3_P	5.25	chlorobenzene; SCRF—ωB97X‐D/6‐311+G(d,p)	[15]
*t*Bu_3_P	5.55	chlorobenzene; SCRF—ωB97X‐D/6‐311+G(d,p)	[15]

Substrates	Electron Affinities [eV]	Conditions	Reference
B(C_6_F_5_)_3_	3.31	chlorobenzene; SCRF—ωB97X‐D/6‐311+G(d,p)	[15]
*p*O_2_C_6_Cl_4_	4.45	chlorobenzene; SCRF—ωB97X‐D/6‐311+G(d,p)	[15]

Substrates	Oxidation Potential [V]	Conditions^[a,b,c,d]^	Reference
PMes_3_	0.784 (anodic); 0.680 (cathodic)	vs. SCE;^[a]^ Pt electrode in butyronitrile in *n*Bu_4_NPF_6_	[38]
*t*Bu_3_P	0.90	CH_3_CN; vs. Fc^+^/Fc	[11]
(Ar)_2_P–P(Ar)_2_ Ar=2,6‐dimethylphenyl	0.795 (anodic); 0.715 (cathodic)	vs. SCE;^[a]^ Pt electrode in butyronitrile in *n*Bu_4_NPF_6_	[38]
(Ar)_2_P‐P(Ar)_2_ Ar=2,4,6‐trimethylphenyl	0.718 (anodic) 0.599 (cathodic)	vs. SCE;^[a]^ Pt electrode in butyronitrile in *n*Bu_4_NPF_6_	[38]
(Ar)_2_P–P(Ar)_2_ Ar=2,4,6‐triethylphenyl	0.592 (anodic); 0.477 (cathodic)	vs. SCE;^[a]^ Pt electrode in butyronitrile in *n*Bu_4_NPF_6_	[38]
P(Ph)_3_	1.03 (irreversible)	vs. Ag/Ag^+^ in DCM;^[b]^ 0.10 M *n*Bu_4_NClO_4_	[45]
P(Mes)_3_	0.41 (reversible)	vs. Ag/Ag^+^ in DCM;^[b]^ 0.10 M *n*Bu_4_NClO_4_	[45]
As(Ph)_3_	1.18 (irreversible)	vs. Ag/Ag^+^ in DCM;^[b]^ 0.10 M *n*Bu_4_NClO_4_	[45]
As(Mes)_3_	0.73 (reversible)	vs. Ag/Ag^+^ in DCM;^[b]^ 0.10 M *n*Bu_4_NClO_4_	[45]
Sb(Ph)_3_	1.05 (irreversible)	vs. Ag/Ag^+^ in DCM;^[b]^ 0.10 M *n*Bu_4_NClO_4_	[45]
Sb(Mes)_3_	0.76 (irreversible)	vs. Ag/Ag^+^ in DCM;^[b]^ 0.10 M *n*Bu_4_NClO_4_	[45]
Me(CH_2_TMS)N‐PhBr	0.23	vs. Fc/Fc^+^;^[c]^ Ag wire in *n*Bu_4_NClO_4_in MeCN RE; Glassy carbon WE; Pt CE	[21]
Me(CH_2_TMS)N‐Ph	0.05	vs. Fc/Fc^+^;^[c]^ Ag wire *n*Bu_4_NClO_4_ in MeCN RE; Glassy carbon WE; Pt CE	[21]
Me_2_N‐PhBr	0.5	vs. Fc/Fc^+^;^[c]^ Ag wire *n*Bu_4_NClO_4_ in MeCN RE; Glassy carbon WE; Pt CE	[21]
B(C_6_F_5_)_3_	*E* ^o^=−1.79 V *E* ^o^=−1.65 V	vs. Fc/Fc^+^ in DCM;^[d]^ vs. Fc/Fc^+^ in difluorobenzene	[46]
	*E* ^o^=−0.64 V	vs. SCE in THF^[a]^	[10]
MesB(C_6_F_5_)_2_	−1.72 (reversible)	vs. Cp_2_Fe^0/+^ in THF;^[e]^ 0.05 M [Bu_4_N] [B(C_6_F_5_)_4_] electrolyte; Pt disk electrodes	[10]
	*E* ^o^=−1.19 V	vs. SCE in THF	
Mes_2_B(C_6_F_5_)	−2.10 (irreversible)	vs. Cp_2_Fe^0/+^ in THF;^e^ 0.05 M [Bu_4_N][B(C_6_F_5_)_4_] electrolyte; Pt disk electrodes	[10]
	*E* ^o^=−1.57 V	vs. SCE in THF	
Mes_3_B	−2.73 (reversible)	vs. Cp_2_Fe^0/+^ in THF;^[e]^ 0.05 M [Bu_4_N] [B(C_6_F_5_)_4_] electrolyte; Pt disk electrodes	[10]
	*E* ^o^=−2.20 V	vs. SCE in THF

[a] SCE: *E*=+0.241 V. [b] Ag/Ag^+^: *E*=+0.197 V. [c] *E*_1/2_ (FeCp_2_^0/+^ vs. SCE)_MeCN_=+0.40 V. [d] *E*_1/2_ (FeCp_2_^0/+^ vs. SCE)_DCM_=+0.46 V. [e] *E*_1/2_ (FeCp_2_^0/+^ vs. SCE)_THF_=+0.56 V; As noted by Jaekle et al.,^[10]^ there is always an element of uncertainty when comparing electrode potential data recorded under different conditions (i.e. solvent, electrolyte, electrodes), hence original data are included herein.

**Scheme 3:**
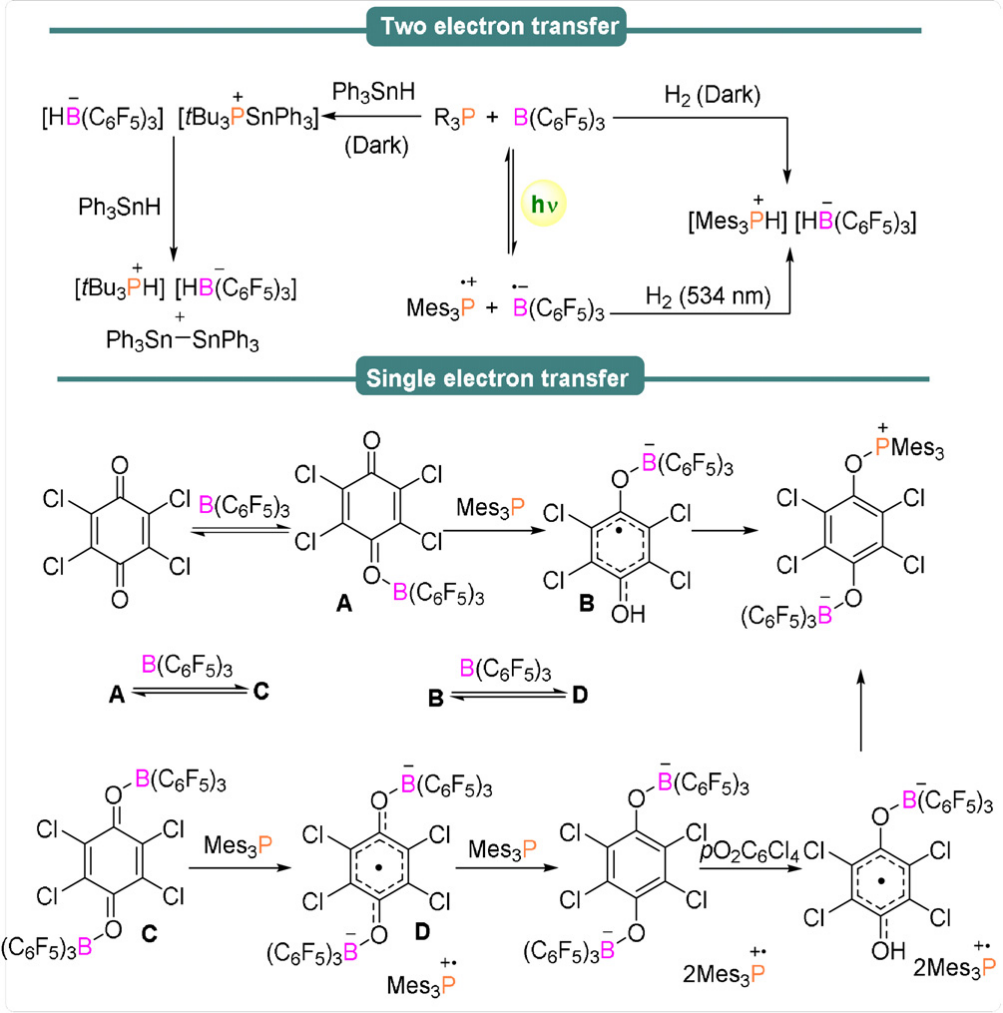
Contrasting reaction pathways between FLPs and FRPs when using *t*Bu_3_P/Mes_3_P and B(C_6_F_5_)_3_.

Stephan et al.^[13]^ reported that an equimolar mixture of Mes_3_P/B(C_6_F_5_)_3_ afforded the radical ion pair [Mes_3_P^.+^][B(C_6_F_5_)_3_]^.−^ but in an insignificantly low concentration because of its short lifetime, determined as 237 ps via transient absorption spectroscopy (TAS) measurements by Slootweg et al.^[14]^ Whilst Stephan et al.^[13]^ postulated a 1 e^−^ transfer process was operative upon reaction of Mes_3_P/B(C_6_F_5_)_3_ with Ph_3_SnH to afford the corresponding phosphonium borate [Mes_3_PH][HB(C_6_F_5_)_3_]/[*t*Bu_3_PH][HB(C_6_F_5_)_3_] (Scheme 3, top), a recent report from Slootweg et al.,^[15]^ reported that the reaction rate of Mes_3_P/B(C_6_F_5_)_3_ (electron‐donor acceptor (EDA) complex at 534 nm) and *t*Bu_3_P/B(C_6_F_5_)_3_ (EDA complex at 400 nm) with H_2_ and Ph_3_SnH remained constant in the dark and during irradiation, indicating that a 2 e^−^ transfer process dominates in these systems.

However, upon a dark reaction of Mes_3_P/B(C_6_F_5_)_3_ with tetrachloro‐1,4‐benzoquinone (TCQ), Slootweg et al. detected several EPR signals, indicating radical formation despite unfavourable electron donor–acceptor characteristics that should prevent thermodynamic SET. This result was explained via weak coordination of B(C_6_F_5_)_3_ to a carbonyl moiety of the quinone acceptor to form a TCQ–B(C_6_F_5_)_3_ adduct with an increased electron affinity, therefore enabling facile SET from the Mes_3_P HOMO. This generates the resulting PMes_3_^.+^/TCQ–B(C_6_F_5_)_3_^.−^ radicals observed via EPR (in addition to an unassigned radical; Scheme 3, bottom).

Evidence for SET between Mes_3_P and Al(C_6_F_5_)_3_ was provided in the form of EPR measurements, which revealed the presence of a doublet resonance with *a*_iso_(^31^P)=238 G (669 MHz) centered on *g*_iso_=2.0089, assigned to the known radical cation Mes_3_P^.+^ (**1**). The corresponding [Al(C_6_F_5_)_3_]^.−^ was not detected in the EPR measurements because of its short lifetime.^[13]^ It is noted that introduction of the bulkier R=SiMe*t*Bu_2_ group has previously facilitated isolation of stable radical anions of R_3_Al^.−^ (**2**) and R_3_Ga^.−^ (**3**), enabling characterization by EPR spectroscopy (see Table 1). The large steric bulk of the R=SiMe*t*Bu_2_ group enforces a planar π‐type radical anion structure, with only small hyperfine couplings arising from the unpaired electron localized in the 3p_*z*_ orbital of the central Group 13 atom.^[16]^

**Scheme 4:**
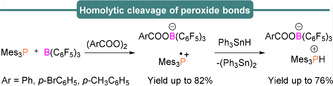
P/B FLP‐mediated homolytic cleavage of peroxides.

The equimolar mixture of the Mes_3_P and B(C_6_F_5_)_3_ FLP was also found to form radical salts [Mes_3_P]^.+^[RCOOB(C_6_F_5_)_3_]^.−^ (R=Ph, *p*‐BrC_6_H_5_, *p*‐CH_3_C_6_H_5_) when reacted with benzoyl peroxide and derivatives (Scheme 4).^[17]^ Again the phosphonium radical cation (**1**) could be observed by EPR spectroscopy. These radical salts further react with Ph_3_SnH as above to produce the salt [Mes_3_PH] [RCOOB(C_6_F_5_)_3_] and (Ph_3_Sn)_2_. Recently, we^[18]^ have demonstrated the same FLP system can be utilized as a powerful metal‐free tool for C–H activation and C−C bond formation which we propose takes place via a single‐electron transfer reaction in which B(C_6_F_5_)_3_ first coordinates to the substrate to initiate the single‐electron transfer. An equimolar mixture of B(C_6_F_5_)_3_ and Mes_3_P in the presence of a fluoro‐substituted benzhydryl ester derivative gave rise to the well characterized isotropic EPR signal of Mes_3_P^.+^ (**1**), resulting from a SET process. In addition, a much weaker and poorly resolved signal was observed centered at *g*_iso_=2.006, whose intensity could be increased upon heating the reaction solution to 70 °C in situ in the EPR cavity. When the 9*H*‐fluorenyl ester was employed in the reaction, a weak EPR signal centered on *g*_iso_=2.0045 with a complex multiplet ^1^H hyperfine pattern was observed.

**Scheme 5:**
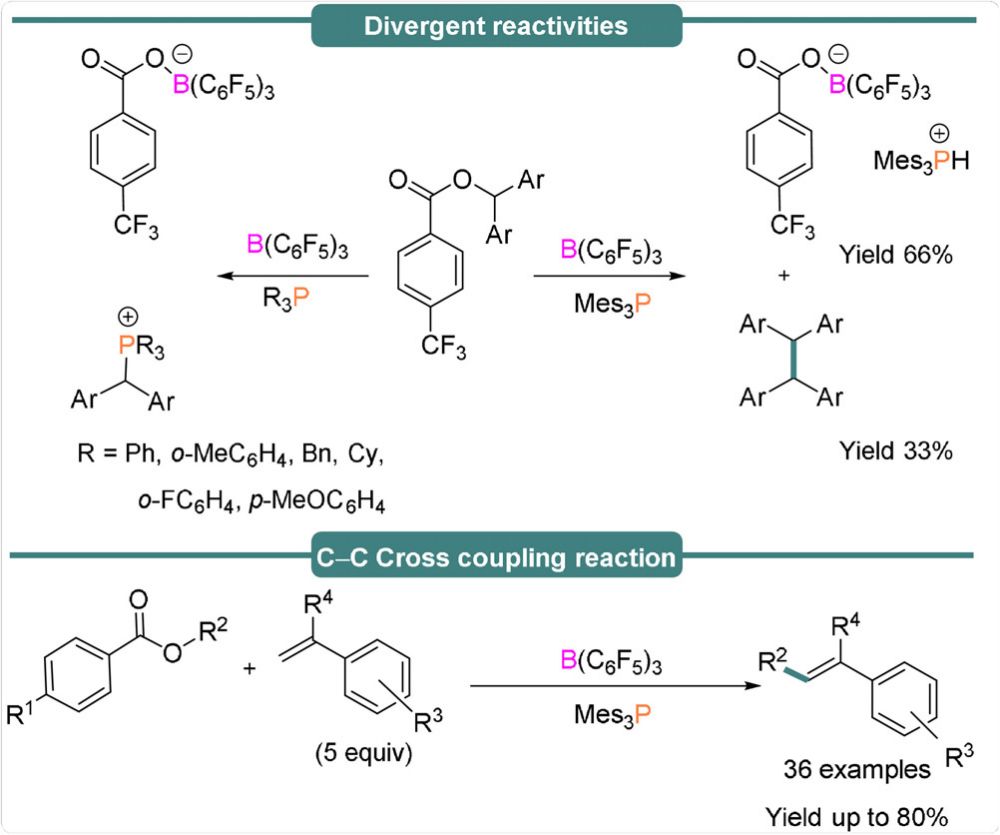
The divergent reactivities of FLPs towards diaryl esters.

Through simulation and comparison to literature reports, this was tentatively assigned to the fluorenyl radical **4**, formed upon FLP‐mediated cleavage of the C(sp^3^)−O bond (see Table 1). In the absence of olefins, the diaryl radicals undergo a homocoupling reaction to yield tetraarylethane derivatives. Interestingly, when other phosphines were used (e.g. *t*Bu_3_P, Ph_3_P) then no homo‐coupling was observed, and phosphonium borate salts resulted (Scheme 5, top). The different reactivities of these phosphines can be explained as a result of their smaller size and higher ionization energies, which leads to formation of the corresponding phosphonium borate salts, which are comparatively more stable than the mesityl phosphine analogue. In the presence of olefins an sp^2^–sp^3^ C–C hetero‐coupling reaction was observed to generate α,β‐substituted olefins (33 examples, yields up to 84 %) (Scheme 5, bottom).

Whilst SET has clearly been evidenced in a series of Mes_3_P/B(C_6_F_5_)_3_ FLP reactions as demonstrated above, the driving force of this process is based on thermodynamic parameters such as ionization potentials, electron affinities and steric bulk of the participating LA/LB. Recent studies from Slootweg et al.,[^14^, ^15^] explored the relative ionization potentials and electron affinities of the Lewis acid and base to explain formation of FRPs. The authors concluded that the large energy gap to create radicals in the archetypal Mes_3_P/B(C_6_F_5_)_3_ system renders thermally activated SET unlikely. Rather, the authors propose that a photoexcitation process akin to those observed in donor–acceptor complexes may be responsible for radical ion generation. The low‐temperature EPR spectrum of violet Mes_3_P/B(C_6_F_5_)_3_ or *t*Bu_3_P/B(C_6_F_5_)_3_ toluene solutions prepared in the dark showed no radical formation by EPR—hence, bringing into question the previous correlations made between purple‐colored solutions observed when using the Lewis base Mes_3_P and the presence of R_3_P^.+^. Subsequent irradiation of these solutions (390–500 nm) led to the observation of two intense EPR signals in both cases. The first broad featureless signal was assigned to the boron radical anion, [B(C_6_F_5_)_3_]^.−^ (**5**).

**Figure 2:**
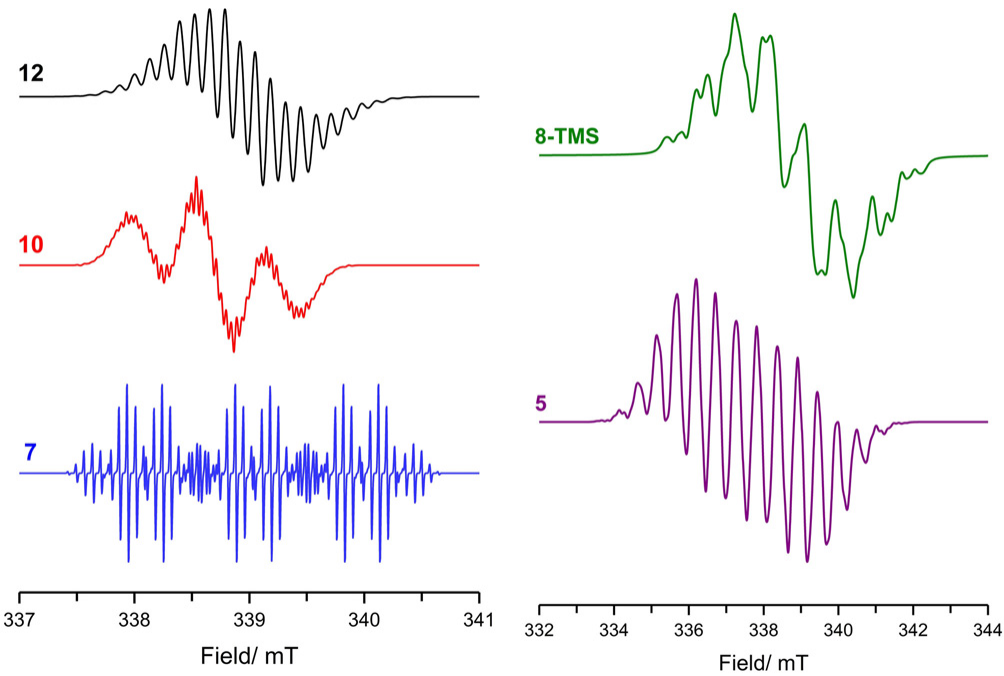
Isotropic CW EPR spectra of **12**, **10** and **7** (left), and **8‐TMS** and **5** (right), simulated using data reported in Table 1.

Whilst no hyperfine coupling was observed for **5** under these experimental conditions, a well‐resolved EPR spectrum for this radical anion has previously been fully reported by Norton et al.,^[19]^ characterized by *g*_iso_=2.0114, *a*_*i*so_(^10, 11^B)=31 MHz, *a*_*i*so_(^19^F_*60*_)=12.94 MHz, *a*_*i*so_(^19^F_*6m*_)=3.66 MHz and *a*_*i*so_(^19^F_*3p*_)=14.9 MHz (Table 1, Figure 2). The second axially symmetric signal was assigned to Mes_3_P^.+^ (**1**) or *t*Bu_3_P^.+^ (**6**), characterized by *g*_∥_=2.0015, *g*_⊥_=2.0055, *A*_∥_(^31^P)=1170 MHz, *A*_⊥_(^31^P)=550 MHz, and *g*_∥_=2.0012, *g*_⊥_=2.0065, *A*_∥_(^31^P)=1365 MHz, *A*_⊥_(^31^P)=580 MHz, respectively. The signal intensity decreased by 25 % 6 mins after cessation of the irradiation (at 30 K), indicating separation of the radical cations and anions in frozen solvent.

**Scheme 6:**
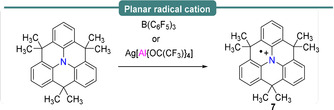
Single‐electron oxidation of an amine by B(C_6_F_5_)_3_.

It is noteworthy that single‐electron transfer from nitrogen Lewis bases to the Lewis acidic borane B(C_6_F_5_)_3_ to afford reactive radical pairs has also been investigated. Wang et al.,^[20]^ reported the one‐electron oxidation of a methylene‐bridged triphenylamine by B(C_6_F_5_)_3_ (Scheme 6). The authors observed a blue solution (*λ*_max_=600 nm) that yielded an EPR spectrum (characterized by *a*_iso_(^14^N)=9.4 G (26.3 MHz); *a*_iso_(^1^H_*3p*_)=3.04 G (8.52 MHz); *a*_iso_(^1^H_*6m*_)=0.71 G (1.99 MHz)), which was assigned to the formation of a stable triphenylamine radical cation (**7**), with spin delocalization across the whole structure (Table 1, Figure 2). An identical spectrum was observed upon reaction with Ag[Al(OC(CF_3_)_3_)_4_] as an alternative to B(C_6_F_5_)_3_. It is noteworthy to mention that the authors did not observe the formation of the boron centered radical anion, which was attributed to the possible decomposition of reactive boron intermediates into various four‐coordinate borates. This work provided the first example of single‐electron oxidation of an organic compound using B(C_6_F_5_)_3_.

**Scheme 7:**
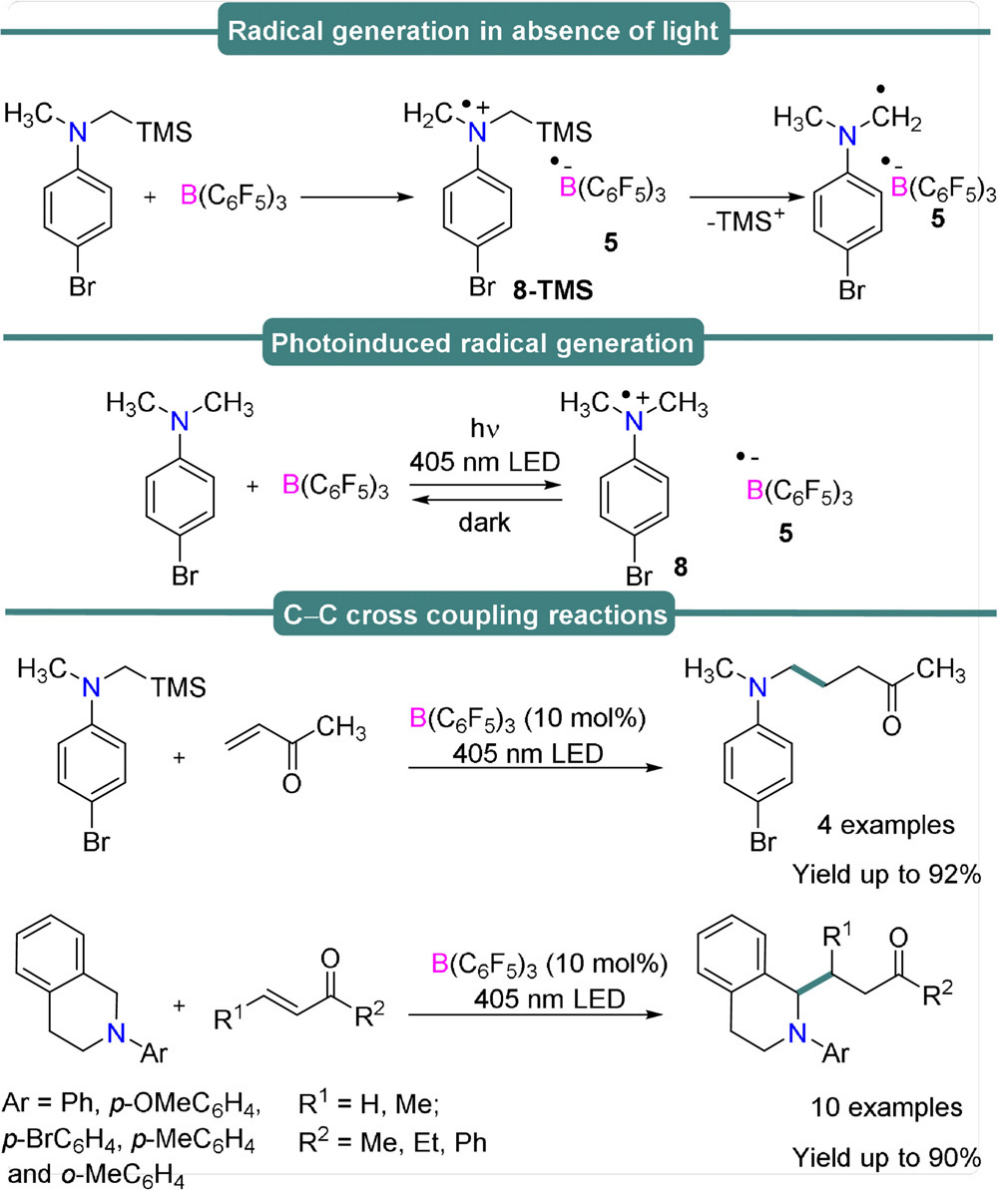
Generation of radical species with or without light (top), and C−C bond formation reaction through single‐electron transfer (bottom).

Very recently, Ooi and co‐workers^[21]^ explored the description of FLPs as EDA complexes in their investigation of single‐electron transfer between the Lewis acidic borane B(C_6_F_5_)_3_ and *N*,*N*‐dialkylaniline Lewis bases including their subsequent catalytic application towards a C−C bond forming reaction (Scheme 7).

*N*,*N*‐Dialkylaniline derivatives were employed for the reaction with B(C_6_F_5_)_3_ in the presence or absence of photoirradiation (405 nm LED light source). Alkyl amines react with B(C_6_F_5_)_3_ to generate reactive α‐aminoalkyl and borane radical pairs, as thoroughly investigated via EPR (Scheme 7, top and center). An equimolar mixture of 4‐bromo‐*N*‐methyl‐*N*‐((trimethylsilyl) methyl)aniline and B(C_6_F_5_)_3_ in CH_2_Cl_2_ at room temperature (Scheme 7, top) yielded an EPR spectrum characterized by *g*_iso_=2.0033, *a*_iso_(^14^N)=23.1, *a*_iso_(^1^H_*m*_)=3.73, *a*_iso_(^1^H_*o*_)=9.68, *a*_iso_(^1^H_*methylene*_)=27.8, *a*_iso_(^1^H_*methyl*_)=20.7 and *a*_iso_(^29^Si)=8.78 MHz, assigned to the radical cation of 4‐bromo‐*N*‐methyl‐*N*‐((trimethylsilyl) methyl)aniline, **8‐TMS** ^.+^ (Table 1, Figure 2). The stability of this radical cation was attributed to hyper‐conjugation at the Si−C bond, as evidenced by the lack of any EPR signal corresponding to the neutral radical formed upon loss of TMS^+^. The formation of the para‐bromo‐*N*,*N*‐dimethylaniline radical cation was not thermally accessible in the dark, but could be photoinduced via a SET process upon irradiation with a 405 nm LED light source. Photoinduced formation of **8** (Scheme 7, middle) was detected via EPR spectroscopy (*g*_iso_=2.0029, *a*_iso_(^14^N)=92.25, *a*_iso_(^1^H_*methyl*_)=60.72, *a*_iso_(^1^H_*o*_)=32.55, *a*_iso_(^1^H_*m*_)=16.12 MHz) (Scheme 7, Table 1), the signal intensity of which was rapidly attenuated after cessation of the irradiation, indicating a back‐electron transfer (BET) process. These findings suggest that the generation and subsequent experimental observation of radical ion pairs is an intricate balance between the energy barriers for SET/BET (which are related to the difference between Lewis acid/base redox potentials, Table 2), and the stability of the radical ion pair (which may be determined by other degradation pathways and which are very active for some of these highly unstable radicals).

The key intermediate of this unique SET process is an EDA complex, and the nucleophilic α‐aminoalkyl radicals generated can be readily exploited to react with electron deficient olefins to make new C−C bonds. In extension of their studies on photo‐generated FRPs, Slootweg et al.,^[14]^ reported that upon varying the Lewis base to incorporate *N*‐based triphenylamine (Ph_3_N) and tri‐*p*‐tolylamine (*p*Tol_3_N), amine radical cations could be observed via EPR spectroscopy under visible light (390–500 nm) conditions (but not in the dark) at room temperature. The isotropic EPR spectrum of *p*Tol_3_N with B(C_6_F_5_)_3_ displayed a broad featureless signal centered at *g*_iso_ ≈2.005, whereas that of Ph_3_N displayed a 3‐line multiplet signal (also at *g*_iso_ ≈2.005), presumably arising from localization of the electron spin density on the ^14^N (*I*=1) nucleus (no hyperfine couplings were reported). These key findings demonstrate that encounter FLP complexes can also be described as electron donor–acceptor complexes which may undergo photo‐induced SET to produce radical pairs.

## 3 Group 13/14 Frustrated Lewis Pairs

**Scheme 8:**
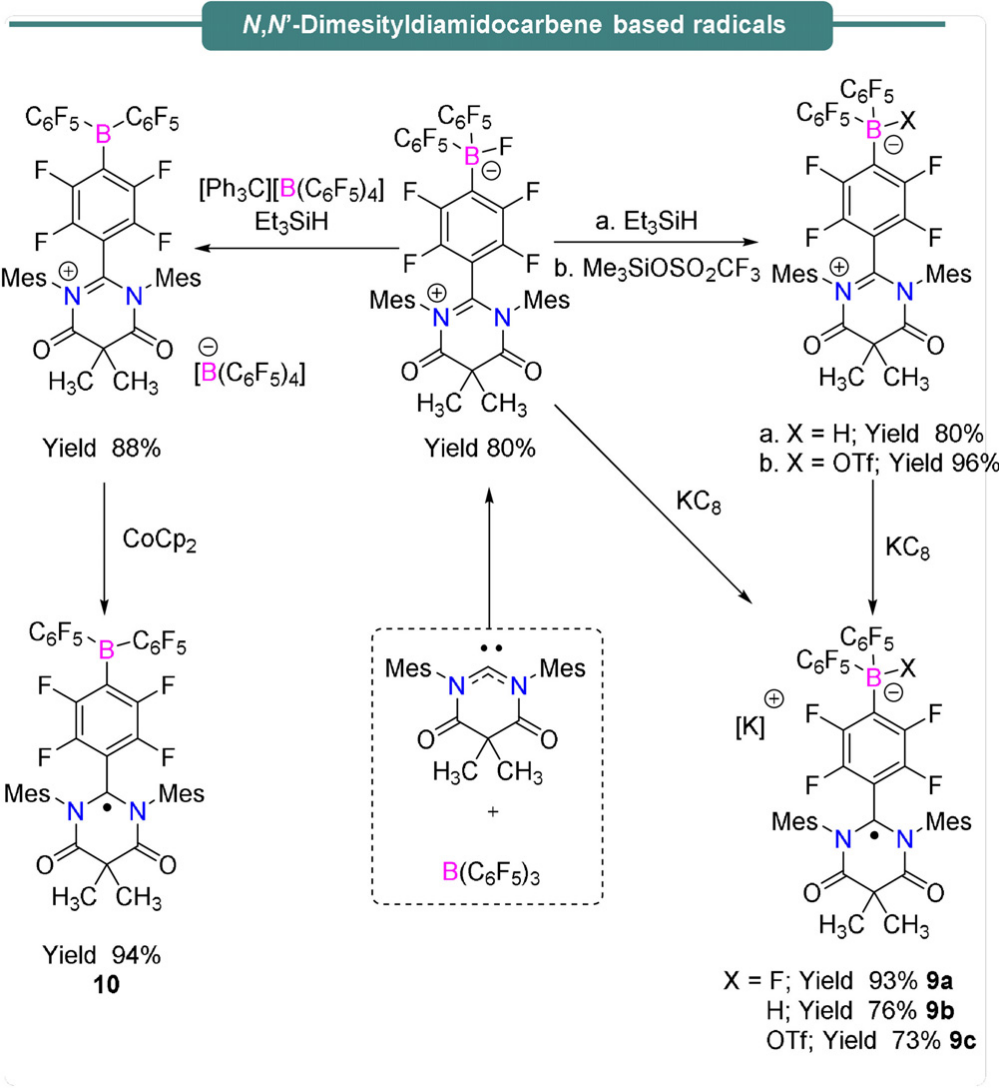
Synthesis of carbon‐based radical species bearing a *N*,*N*′‐dimesityldiamidocarbene group.

The exciting preliminary outcomes from frustrated Lewis pair chemistry has drawn considerable interest in main group chemistry. Radical chemistry of FLPs containing boron Lewis acids and Group 14 Lewis bases such as carbenes or germylene have also been studied. Stephan et al.,^[22]^ demonstrated the reactivities of Lewis acidic boranes towards the nucleophilic carbene *N*,*N*′‐dimesityldiamidocarbene (DAC) (Scheme 8). The reaction between DAC and B(C_6_F_5_)_3_ in benzene afforded a crystalline solid Me_2_C(CONMes)_2_CC_6_F_4_BF(C_6_F_5_)_2_ in 80 % yield via nucleophilic attack of DAC to one of the *para* positions of a C_6_F_5_ ring of B(C_6_F_5_)_3_, followed by fluoride transfer to the boron center.

Further treatment of the above crystalline compounds with Et_3_SiH or TMSOTf afforded zwitterionic Me_2_C(CONMes)_2_CC_6_F_4_BX (C_6_F_5_)_2_ (X=H or OTf) as yellow powders in 80 % and 96 % yield, respectively. Reduction of these compounds where X=F, H, or OTf with KC_8_ afforded the corresponding radical anions [Me_2_C(CONMes)_2_CC_6_F_4_BX(C_6_F_5_)_2_]^.−^, (**9 a**–**c**), which all gave rise to complex hyperfine structure in their corresponding isotropic EPR signals centered at *g*_iso_=2.022 (**9 a**) and *g*_iso_=2.003 (**9 b**,**c**) (see Table 1 for details). Alternatively, if the boron‐bound fluoride ion from the zwitterionic Me_2_C(CONMes)_2_CC_6_F_4_BF(C_6_F_5_)_2_ is abstracted using [Et_3_Si][B(C_6_F_5_)_4_], the formation of the [Me_2_C(CONMes)_2_CC_6_F_4_B(C_6_F_5_)_2_]^+^[B(C_6_F_5_)_4_]^−^ ion pair results in 88 % yield. The isotropic EPR spectrum of this complex following treatment with CoCp_2_, centered at *g*_iso_=2.004 (Figure 2, Table 1) was assigned to the neutral radical **10** (Scheme 8, Figure 2). The authors noted that the isolable nature of these radicals contrasts with the transient nature of the thermally unstable [B(C_6_F_5_)_3_]^.−^ (**5**) radical anion, which was attributed to stabilization of the radical character by distribution of the unpaired electron over the C_6_F_4_ linker group and DAC substituent. These results provided rare examples of isolable electrophilic boron centers and spontaneous formation of radicals with Lewis acid/base combinations, which may be further utilized in synthetic FRP chemistry.

**Scheme 9:**
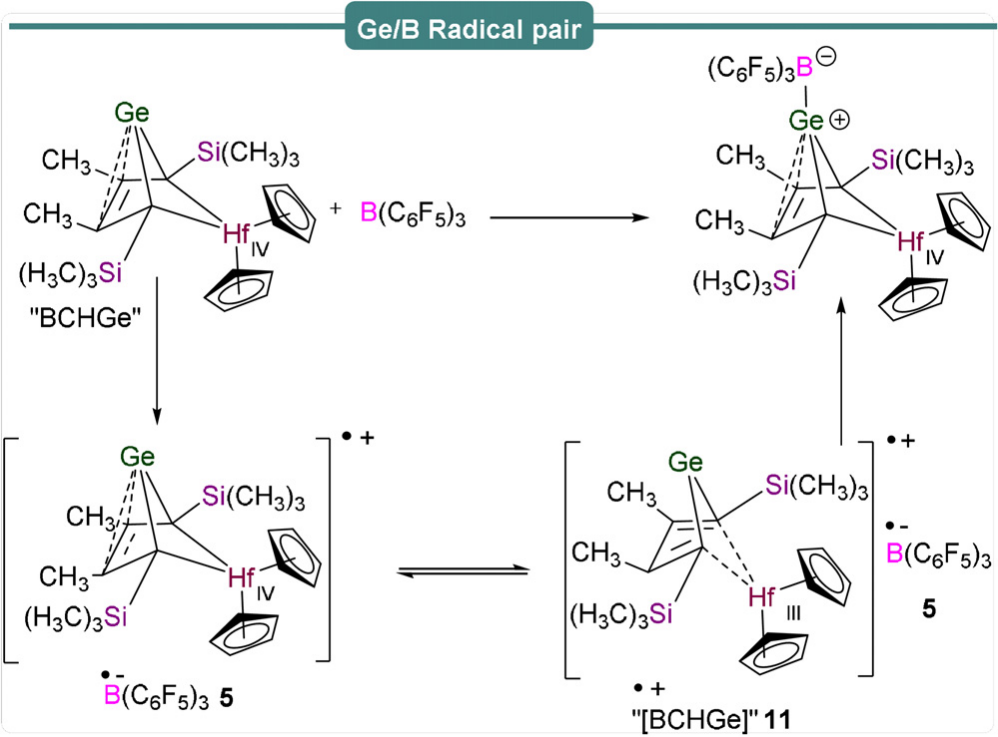
Generation of radical pair species bearing Ge and B.

The generation of FRPs as opposed to formation of the polar products LA^−^/LB^+^ relies on prevention of spin‐pairing, which can either be facilitated through steric bulk, or valence isomerization of the products formed after the initial SET process. This was recently exemplified by Müller et al.,^[23]^ in an investigation of SET in a Ge/B FLP, employing a hafnocene‐based germylene as the Lewis base in the reaction with B(C_6_F_5_)_3_ to afford a B−Ge‐bonded species (Scheme 9). The EPR spectrum of a toluene solution of B(C_6_F_5_)_3_ and a bicyclohexane‐germylene, labelled “BCHGe”, revealed an intense singlet centered at *g*_iso_=1.9881 surrounded by weak satellite features originating from coupling to spin‐active hafnium nuclei (*I*(^177^Hf)=7/2, 18.6 % abundance; *I*(^179^Hf)=9/2, 13.6 % abundance) characterized by *a*_iso_(Hf)=85 G (236.5 MHz). This signal, which decayed to zero over 3.5 h, was attributed to a Ge^..^/Hf^III^‐based radical cation, labelled “[BCHGe]^.+^”, **11** (Scheme 9), formed upon oxidation of BCHGe in which the total spin density is mainly localized on the d_z_^2^ orbital of the hafnium atom, with minimal contribution of the lone pair on germanium.

## 4 Group 14/15 and 14/14 Frustrated Lewis Pairs

**Scheme 10:**
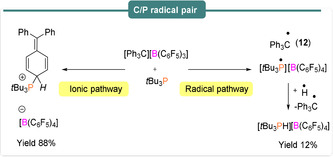
Generation of carbon‐ and phosphorus‐based radical pairs.

Radical behaviour of both trityl cations and silylium cations, isoelectronic and isolobal to B(C_6_F_5_)_3_, have also been investigated when used as the Lewis acid component of an FLP with carbenes, germylenes or phosphines acting as the Lewis base. In Section 2, the generation of carbon‐based radicals was observed in the reactions of the Mes_3_P/B(C_6_F_5_)_3_ FLP with diaryl esters.^[18]^ Although it was already known that two‐electron nucleophilic addition of *t*Bu_3_P to the *para* position of the trityl borate [Ph_3_C][B(C_6_F_5_)_4_] leads to the formation of the cyclohexa‐2,5‐diene‐phosphenium [B(C_6_F_5_)_4_] salt (Scheme 10, left),^[24]^ subsequent evidence^[25]^ showed that a single‐electron pathway was also operational (Scheme 10, right). Reaction in benzene or chlorobenzene solution yielded the characteristic EPR spectra of the Ph_3_C^.^ trityl radical (**12**), characterized by *g*_iso_=1.999, and a rich hyperfine structure of *a*_iso_(^1^H_*60*_)=7.27 MHz, *a*_iso_(^1^H_*6m*_)=3.08 MHz and *a*_iso_(^1^H_*3p*_)=7.83 MHz originating from the ring protons (Figure 2). The radical cation *t*Bu_3_P^.+^ however could not be observed by EPR spectroscopy due to its short lifetime.

**Scheme 11:**
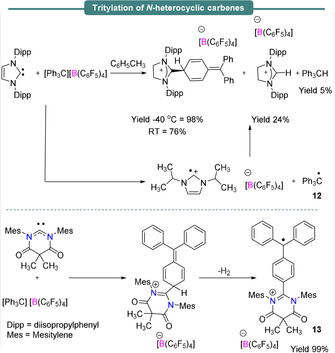
Formation of carbon‐based radicals.

Instead of phosphines as the Lewis base, carbenes have also been employed in combination with the same Lewis acid. Recently, single‐electron transfer reactions generating C‐based radicals in classical Lewis pairs have been observed and highlighted by Severin et al.^[26]^ In the reaction between the carbene 1,3‐bis(2,6‐diisopropylphenyl) imidazol‐2‐ylidene (IDipp) and [Ph_3_C][B(C_6_F_5_)_4_], a single‐electron transfer from the carbene to the trityl borate salt afforded the persistent radical [Ph_3_C]^.^ (**12**), as characterized by EPR spectroscopy. (Scheme 11, top). As a series of colour changes were observed during the reaction, it was further probed via UV/Vis spectroscopy.

At the beginning of the reaction, a gradual decrease of an absorption band at 438 nm was observed, assigned to the trityl cation, and simultaneously a band at 343 nm assigned to the trityl radical began to grow. A weak band at 591 nm observed at the start of the reaction, which disappeared over time, provided evidence of the IDipp^.+^ radical cation, which was not identified via EPR spectroscopy due to its high reactivity. These complementary techniques facilitated full mechanistic insight.

Stephan et al.,^[22]^ have also investigated the reactivities of the same trityl salt [Ph_3_C][B(C_6_F_5_)_4_] with the nucleophilic DAC carbene described earlier (Scheme 11, bottom). Nucleophilic attack of DAC at one of the *para*‐positions of the trityl cation was observed, followed immediately with H_2_ evolution to afford a cationic radical [Me_2_C(CONMes)_2_C C_6_H_4_CPh_2_C]^.+^ (**13**) (Scheme 11, bottom). The EPR spectrum of this species gave a *g*_iso_ value of 1.993, with simulation of the hyperfine coupling consistent with delocalization of the radical over the trityl moiety.

**Scheme 12:**
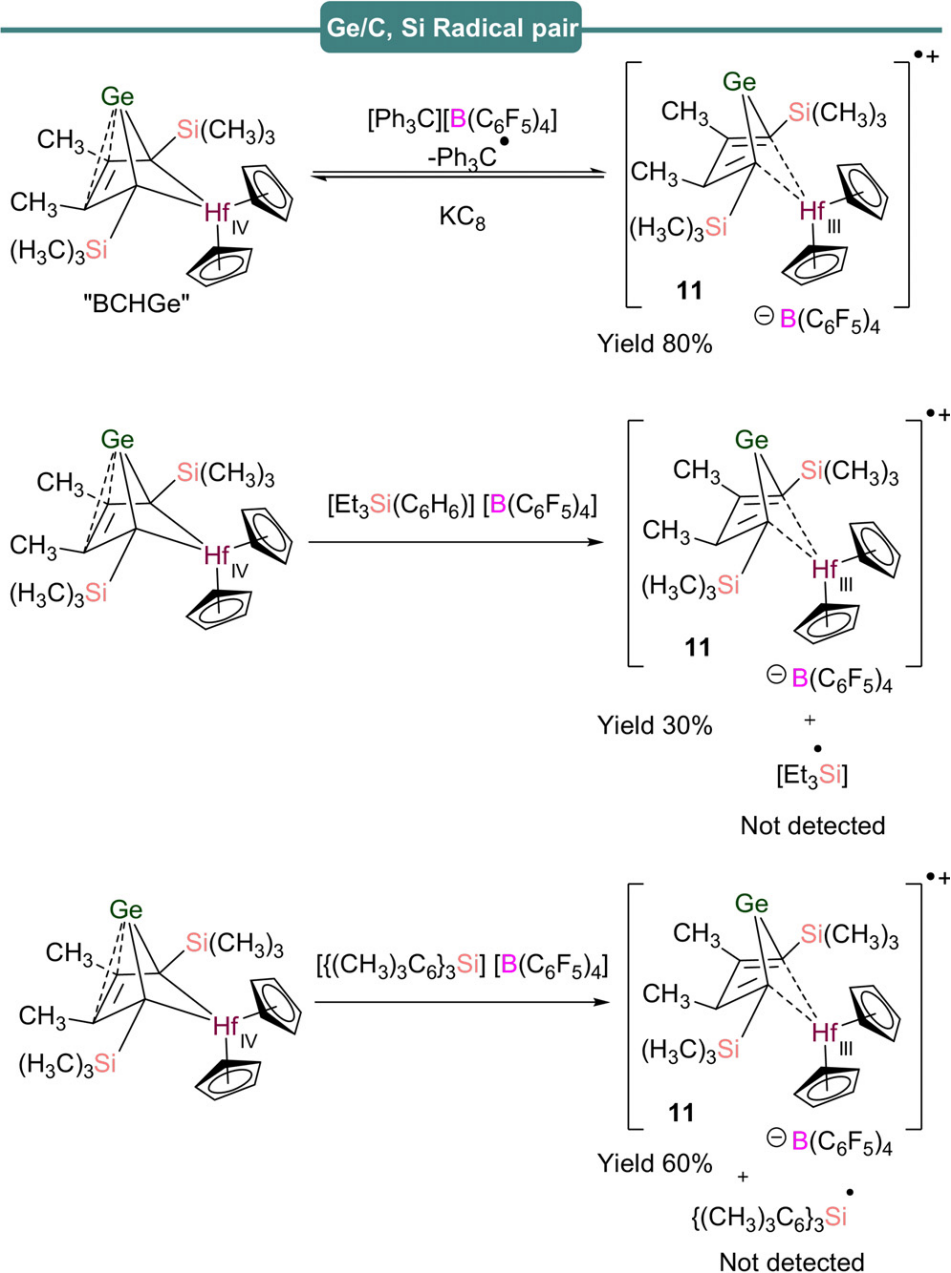
Germanium‐ and carbon‐/silicon‐based radical pairs.

Moving down Group 14, reaction of the BCHGe species described in Section 3 with the trityl cation [Ph_3_C][B(C_6_F_5_)_4_] and separation of the biphasic mixture into products also yielded EPR active solutions (Scheme 12, top).^[23]^ The EPR spectrum of the organic phase revealed the presence of the trityl radical Ph_3_C^.^ (**12**) characterized by a rich hyperfine structure originating from the ring protons, centered on *g*_iso_=1.9980 (see Table 1), whereas [BCHGe]^.+^ (**11**) was detected in the polar phase. Similarly, when a silyl arenium borate [Et_3_Si(C_6_H_6_)][B(C_6_F_5_)_4_] or a silylium borate [(Me_5_C_6_)_3_Si][B(C_6_F_5_)_4_] was treated with BCHGe, EPR signals for **11** were observed but no EPR active signals for silyl‐centered radicals were detected.

**Scheme 13:**
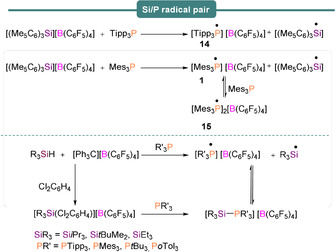
Reaction of silylium borate with Tipp_3_P and Mes_3_P (top), and the synthesis of solvent‐stabilized silylium borates and subsequent generation of phosphorus‐ and silicon‐based radicals (bottom).

Despite the lack of observation of silyl radicals in the hafnocene‐based germylene derivative, Müller et al.,^[23]^ were able to evidence the utility of single‐electron transfer reactions for different combinations of silylium ion/phosphine Lewis pairs (Scheme 13). For example, when Tipp_3_P (Tipp=2,4,6‐triisopropylphenyl) was treated with [(Me_5_C_6_)_3_Si][B(C_6_F_5_)_4_] the radical salt [Tipp_3_P]^.^[B(C_6_F_5_)_4_] ([**14**][B(C_6_F_5_)_4_]) resulted along with the silyl radical [(Me_5_C_6_)_3_Si]^.^ (Scheme 13, top). The EPR spectrum of the reaction mixture revealed a doublet signal centered on *g*_iso_=2.0015 with *a*_iso_(^31^P)=238 G (667 MHz), characteristic of the P‐based Tipp_3_P^.+^ radical cation (**14**). Again, direct EPR evidence for the triarylsilyl radicals was not obtained as a result of their very short lifetimes. Radical scavenging using TEMPO ((2,2,6,6‐tetramethylpiperidin‐1‐yl)oxyl) free radical) and cyclohexadiene also proved inconclusive. Use of the less bulky phosphine Mes_3_P with [(Me_5_C_6_)_3_Si][B(C_6_F_5_)_4_] yielded multiple signals in the EPR spectrum. The authors assigned one of these to Mes_3_P^.+^ (**1**) and upon close inspection of their results, we suggest that the unassigned features in their EPR spectra are due to a [P(Mes)_*n=2,3*_]_2_^.+^ dimer, formed upon rapid reaction of the monomer radical cation with a second molecule of phosphine to produce the dimer cation radical. The EPR spectrum of [((Mes)_2_)_2_P^.+^, **15**]^[27]^ has previously been reported (Scheme 13 top, and Figure 1), and it is noted that previous literature examples of phosphine dimer cation radicals of divalent (R_2_P)_2_^.+^ and trivalent (R_3_P)_2_^.+^ systems yield very similar EPR spectra, dominated by the phosphorus hyperfine.

SET was also observed by these authors with the use of trialkylsilylium ions, stabilized in *o*‐C_6_H_4_Cl_2_ (Scheme 13, bottom). Thus, reaction of *o*‐C_6_H_4_Cl_2_‐stabilized silylium borates [R_3_Si][B(C_6_F_5_)_4_] (R_3_Si=*i*Pr_3_Si, *t*BuMe_2_Si, Et_3_Si) with different phosphines R′_3_P (R′=Tipp, Mes, *t*Bu, *o*‐Tol) were tested and the SET reactions between the silylium ion/phosphine were studied (Scheme 13, bottom). The experimental results indicated that the radical mechanism is not restricted to sterically encumbered triarylsilylium ion‐based FLPs but may also apply to solvent‐ stabilized trialkylsilylium ions. Use of Lewis acids with strong electron affinities is recommended to induce one‐electron oxidation to facilitate novel radical reactions.

## 5 FLP‐NO Radicals

**Scheme 14:**
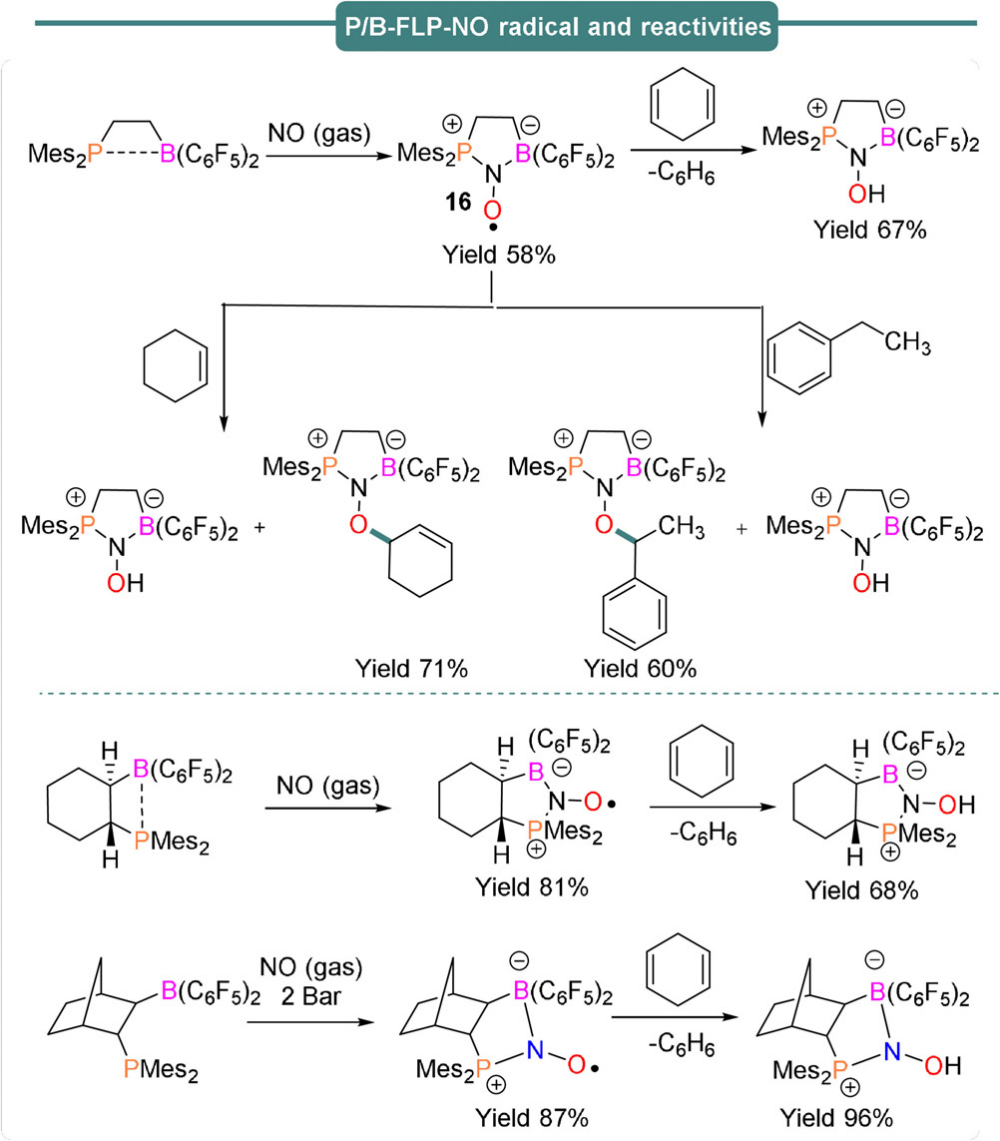
Synthesis and reactivity of intramolecular FLP‐NO radicals.

Development of facile and mild synthetic strategies for the synthesis of various aminoxyl (nitroxyl) radicals are exciting as these types of persistent radical compounds have diverse applications in chemistry as well as biological sciences.^[28]^ The formation of sterically encumbered, persistent aminoxyl radicals has been investigated with an emphasis on elucidating their stability and reactivity. Intramolecular frustrated phosphino‐borane Lewis pairs have been found to be reactive towards NO and to afford FLP‐NO *N*‐oxyl radicals.^[29]^ *N*,*N*‐cycloaddition of C2‐bridged intramolecular P/B frustrated Lewis pairs with nitric oxide has been demonstrated by Erker et al.^[30]^ in 2011. The authors observed that an intramolecular ethylene‐bridged FLP system Mes_2_PCH_2_CH_2_B(C_6_F_5_)_2_ can readily react in situ with 1 equiv nitric oxide (NO_gas_) to form the persistent heterocyclic *N*‐oxyl radical P/B‐FLP‐NO^.^ (**16**) in 58 % yield (Scheme 14, top). Although nitric oxide is inert towards H‐atom abstraction (HNO bond strength 47 kcal mol^−1^), the cyclic P/B‐FLP‐NO^.^ radical species (**16**) was found to be active towards H‐atom abstraction from stronger C−H bonds when reacted with for example, 1,4‐cyclohexadiene or ethylbenzene, to afford the diamagnetic P/B‐FLP‐NOH and/or P/B‐FLP‐NOR species (Scheme 14, top).

**Scheme 15:**
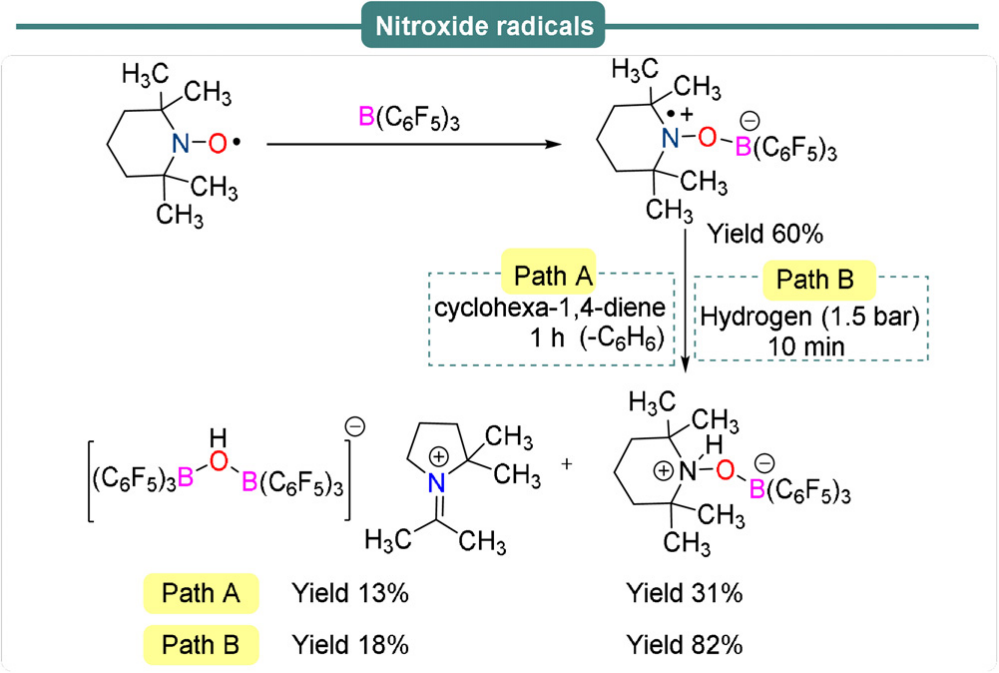
Formation of persistent nitroxide radicals under frustrated Lewis pair conditions.

In both cases the P/B‐FLP‐NOH species formed along with P/B‐FLP‐NOR in a 1:1 ratio. The presence of a single electron in the P/B‐FLP‐NO^.^ **16** adduct was confirmed via EPR spectroscopy, revealing *g*_iso_=2.0089, *a*_iso_(^14^N)=18.5, *a*_iso_(^31^P)=48.5 and *a*_iso_(^11^B)=9.1 MHz. Similar reactivity is observed when an intramolecular cyclohexylene‐bridged P/B FLP or an intramolecular norbornane‐bridged P/B FLP are allowed to react with NO (Scheme 14, bottom). In both cases, formation of the persistent FLP‐NO^.^ aminoxyl radical species was observed.[^31^, ^32^, ^33^] As in the first example, the oxygen‐centered aminoxyl radicals were found to be highly reactive and both undergo H‐atom abstraction with 1,4‐cyclohexadiene to give the diamagnetic FLP‐NOH product. DFT and kinetic studies, along with reaction monitoring through multinuclear NMR (^1^H, ^19^F, ^31^P), have been employed to highlight the reaction mechanism. Formation of reactive radical intermediates was also monitored using UV/Vis spectroscopy and detailed EPR studies have been performed to confirm the formation of those radicals.^[33]^ The stable TEMPO radical has also been observed to act as a Lewis base towards strongly Lewis acidic B(C_6_F_5_)_3_. The TEMPO‐B(C_6_F_5_)_3_ adduct was found to be in equilibrium with the unquenched form and can thus act as an FLP (Scheme 15). Indeed, while the TEMPO^.^ radical is inert toward dihydrogen, the TEMPO/B(C_6_F_5_)_3_ FLP system was found to be active towards dehydrogenation of 1,4‐cyclohexadiene (Path A) as well as dihydrogen activation under mild reaction conditions (Path B).^[34]^

**Scheme 16:**
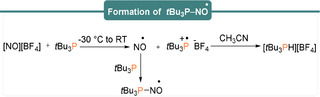
Single‐electron oxidation of *t*Bu_3_P by a nitrosonium borate salt.

High reactivities of FLPs towards NO were demonstrated to account for the formation of reactive aminoxyl radicals. On the other hand, Slootweg and co‐workers^[11]^ explored the formation of the NO^.^ radical using the nitrosonium salt [NO][BF_4_] and *t*Bu_3_P. Single‐electron transfer between *t*Bu_3_P and the nitrosonium salt [NO][BF_4_] in acetonitrile generated [HP*t*Bu_3_][BF_4_] as the major product (Scheme 16).

The formation of this product was proposed to proceed through the formation of the radical intermediate [*t*Bu_3_P]^.+^[BF_4_]^−^ and NO^.^. The radical salt readily abstracts a proton from the solvent to form the phosphonium borate product, whereas EPR studies suggested that the NO^.^ generated reacts with *t*Bu_3_P to give *t*Bu_3_P‐NO^.^.

## 6 Conclusion

Frustrated Lewis pair chemistry has gained considerable interest because of its unique chemical reactivities particularly in small molecule activation. Extensive investigation on their reactivity via single‐electron transfer, and the resulting structural and bonding properties, have revealed a new class of reactivities coined frustrated radial pair (FRP) chemistry. The unique behavior of FLPs/FRPs has successfully been employed in catalysis and synthetic organic chemistry, even using catalytic conditions. Providing complementarity to transition metal catalysis, FLP/FRP chemistry has rapidly garnered considerable interest from the scientific community, for which there are ample opportunities for future development. In particular, whilst it is noted that the operation of SET has currently only been experimentally observed with a small number of LA/LB pairs, it is now understood that FRP generation may be accessed for any combination of LA/LB via either thermochemical or photochemical routes, hence the generation of radical ion pairs as reactive species must continue to be further investigated in order to fully exploit their utility in organic synthesis.

As reviewed herein, the application of EPR spectroscopy is fundamental to the characterization of FRPs, via detection of short‐lived radical intermediates generated under a broad range of experimental conditions, as direct experimental evidence of single‐electron transfer events. The relative redox potentials of the LA/LB, the propensity for formation of electron‐donor complexes and back donation of electrons, and the requirement for photochemical activation are all topics that must be further explored, experimentally and with supporting computational calculations. Whilst outside the immediate focus of this contribution, the reader is encouraged to refer to the excellent reviews detailing the thermodynamic and kinetic factors governing photoelectron transfer catalysis in transition metal (in)organic chemistry,^[35]^ and redox processes in main group systems for further discussion.^[36]^ Overall, this minireview demonstrates the synthesis of several frustrated radical pairs, which we believe opens the potential for new modes of reactivity.

## Conflict of interest

The authors declare no conflict of interest.

## Biographical Information

*Dr. Ayan Dasgupta received his PhD degree from IIT Madras (India) in 2016 where he worked under the supervision of Prof. S. Sankararaman. During this PhD he worked with Prof. H. Hopf as an Indigo exchange student at TU Braunschweig (Germany) in 2013. He later joined TU Munich (Germany) as a postdoctoral research associate in 2017 and worked on hypervalent iodine catalysis under the supervision of Prof. T. Gulder. Ayan joined the Melen research group as a postdoctoral research associate in 2018. His research focus is on the use of main group elements as catalysts in organic synthesis*.

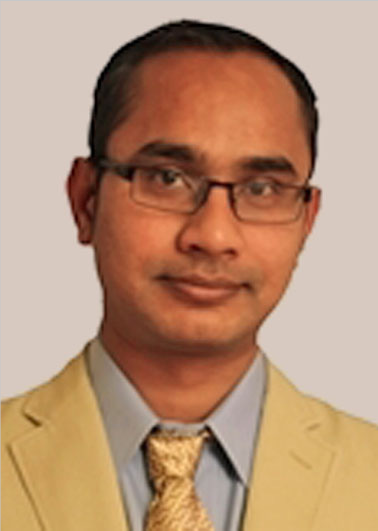

## Biographical Information

*Dr. Emma Richards studied Natural Sciences at the University of Bath (UK)*, *prior to being awarded her PhD from Cardiff University (UK) in 2007. She was appointed to an independent position at Cardiff University in 2015 and is now a Lecturer in Physical Chemistry. Her research involves utilizing EPR spectroscopy and associated hyperfine techniques to identify transient open‐shell intermediates that enable determination of reaction mechanisms. Her broad research interests span homogeneous and heterogeneous catalysis focussing on the electronic characteristics that direct catalytic events. Emma is co‐author of the popular OUP Oxford Chemistry Primer “Electron Paramagnetic Resonance”*.

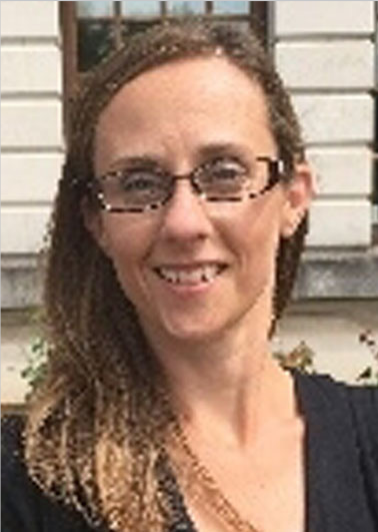

## Biographical Information

*Dr. Rebecca Melen studied for her PhD degree at the University of Cambridge (UK). Following Postdoctoral studies in Toronto (Canada) and Heidelberg (Germany), she took up a position at Cardiff University (UK) in 2014 where she is now a Reader in Inorganic Chemistry. In 2018, she was awarded an EPSRC early career fellowship and she was the 2019 recipient of the RSC Harrison–Meldola Memorial Prize. Her research interests include diverse aspects of main group reactivity and catalysis, including the applications of main group chemistry in organic synthesis*.